# A New Body Shape Index Predicts Mortality Hazard Independently of Body Mass Index

**DOI:** 10.1371/journal.pone.0039504

**Published:** 2012-07-18

**Authors:** Nir Y. Krakauer, Jesse C. Krakauer

**Affiliations:** 1 Department of Civil Engineering, The City College of New York, New York, New York, United States of America; 2 Middletown Medical, Middletown, New York, United States of America; Tulane School of Public Health and Tropical Medicine, United States of America

## Abstract

**Background:**

Obesity, typically quantified in terms of Body Mass Index (BMI) exceeding threshold values, is considered a leading cause of premature death worldwide. For given body size (BMI), it is recognized that risk is also affected by body shape, particularly as a marker of abdominal fat deposits. Waist circumference (WC) is used as a risk indicator supplementary to BMI, but the high correlation of WC with BMI makes it hard to isolate the added value of WC.

**Methods and Findings:**

We considered a USA population sample of 14,105 non-pregnant adults (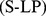) from the National Health and Nutrition Examination Survey (NHANES) 1999–2004 with follow-up for mortality averaging 5 yr (828 deaths). We developed A Body Shape Index (ABSI) based on WC adjusted for height and weight:
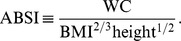

ABSI had little correlation with height, weight, or BMI. Death rates increased approximately exponentially with above average baseline ABSI (overall regression coefficient of 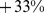 per standard deviation of ABSI [95% confidence interval: 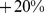
–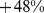]), whereas elevated death rates were found for both high and low values of BMI and WC. 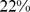 (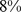
–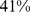) of the population mortality hazard was attributable to high ABSI, compared to 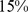 (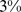
–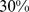) for BMI and 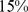 (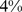
–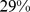) for WC. The association of death rate with ABSI held even when adjusted for other known risk factors including smoking, diabetes, blood pressure, and serum cholesterol. ABSI correlation with mortality hazard held across the range of age, sex, and BMI, and for both white and black ethnicities (but not for Mexican ethnicity), and was not weakened by excluding deaths from the first 3 yr of follow-up.

**Conclusions:**

Body shape, as measured by ABSI, appears to be a substantial risk factor for premature mortality in the general population derivable from basic clinical measurements. ABSI expresses the excess risk from high WC in a convenient form that is complementary to BMI and to other known risk factors.

## Introduction

According to the World Health Organization (WHO), overweight and obesity are increasing in prevalence and rank fifth as worldwide causes of death among risk factors, behind high blood pressure, tobacco use, high blood glucose, and physical inactivity. In high and middle income countries, where the prevalence of overweight and obesity among the adult population already exceeds 50%, overweight and obesity occupy third place as risk factors causing death, behind high blood pressure and tobacco use. WHO defined overweight as body mass index (BMI; weight divided by 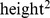) at or above 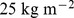, with obesity defined as 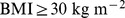 [1]. Guidelines published by the USA National Institutes of Health, using the same definition, considered that overweight and obesity are the second leading cause of preventable death in the USA, behind smoking [2].

These BMI-based obesity guidelines have been accompanied by doubt as to the validity of BMI as an indicator of dangerous obesity. BMI does not distinguish between muscle and fat accumulation [3]–[6], and there is evidence that whereas higher fat mass is associated with greater risk of premature death, higher muscle mass reduces risk [7]. As well, BMI does not distinguish between fat locations, when central or abdominal fat deposition is thought to be particularly perilous [8]–[11]. Waist circumference (WC) has emerged as a leading complement to BMI for indicating obesity risk. A number of studies have found that WC predicted mortality risk better than BMI [12]–[17]. A recent WHO report summarized evidence for WC as an indicator of disease risk, and suggests that WC could be used as a alternative to BMI [18].

A key limitation, mentioned in the WHO report, of using WC as a proxy for abdominal fat distribution is that it is sensitive to body size (height and weight) as well as to fat percentage and distribution. In fact, WC is highly correlated with BMI, to the extent that differentiating the two as epidemiological risk factors can be difficult [19]. According to a consensus statement on the clinical usefulness of WC [20], “Further studies are needed to establish WC cut points that can assess cardiometabolic risk, not adequately captured by BMI and routine clinical assessments.” Scaling WC via allometric analysis to produce a quantity that is independent of BMI offers one means of separating the impact on health of body shape (degree of central bulge, presumably correlating with abdominal fat deposits) from that of body size (as measured by height, weight, and BMI). In this paper, our objectives are (1) develop A Body Shape Index (ABSI) based on WC that is approximately independent of height, weight, and BMI; and (2) evaluate ABSI as a predictor of mortality across age, sex, ethnicity, and BMI categories in a population sample, compared to the conventional predictors BMI and WC.

## Methods

### Description of Data

We employed public-use releases of baseline interview and medical examination and mortality outcome data from the National Health and Nutrition Examination Survey (NHANES) 1999–2004. NHANES 1999–2004 was designed to sample the civilian noninstitutionalized USA population using a cluster approach. Mexicans and blacks, people 12–19 years of age and 60 years or older, low-income whites, and pregnant women were oversampled to better understand the health status of these groups. The survey included a home interview followed by a physical examination at a mobile examination center. Mortality outcomes based on the National Death Index were available through the end of 2006, representing 2–8 years of follow-up. NHANES 1999–2004 was approved by the National Center for Health Statistics (NCHS) Research Ethics Review Board under Protocol #98–12, and written informed consent was obtained from participants [21].

Basic demographic variables included baseline age (because of privacy concerns, this was given as 85 for all those 85 or older), sex, and ethnicity (given as Mexican, other Hispanic, white, black, or other). Body measurements including height, weight, and waist circumference were obtained by trained health technicians following standardized procedures. Standing height was measured using a digital stadiometer with a fixed vertical backboard and an adjustable head piece. Weight in an examination gown was measured on a digital scale. Waist circumference at the end of a normal exhalation was measured to the nearest 0.1 cm with a steel tape positioned just above the uppermost lateral border of the ilium. All instruments were calibrated following uniform protocols. These body measurements were generally not taken for people confined to wheelchairs [22].

We considered adults (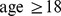) with height, weight, and waist circumference measurements, excluding women determined to be pregnant by self-report or urine pregnancy test. Of the 14,123 individuals meeting these criteria, 14,105 had valid mortality follow-up data. The follow-up period for those remaining alive averaged 4.8 yr, and there were 828 deaths.

Additional variables we considered were smoking status (coded as yes if reported smoking cigarettes in last 5 days or smoking ‘every day’ or ‘some days’), diabetes status (coded yes if reported a diabetes diagnosis or taking insulin or diabetes pills), and systolic and diastolic blood pressure and serum total and HDL cholesterol from the medical examination. A total of 12,044 individuals in our sample had all these variables available, with 647 deaths during follow-up.

We used the NHANES mobile examination center sample weights, which adjust for targeted oversampling and nonresponse, as well as information on which cluster each sampled individual belonged to, following the analytic guidance provided by NCHS [23]. Thus, the average ABSI values and risk estimates we compute can be taken to hold for the wider nonpregnant adult USA population insofar as NHANES was successful in sampling it. Looking at the consistency of responses across subgroups within the sample can also provide some guidance on the wider applicability of our results.

### Construction of the Body Shape Index

We performed linear least-squares regression on 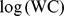 as a function of 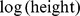 and 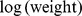 for the entire nonpregnant adult sample. (Pregnant woman averaged bigger WC for a given height and weight.) Expressing WC and height in m and weight in kg, the results were.
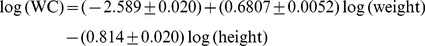
(1)


(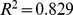), where the given uncertainties are standard errors. Approximating the obtained regression coefficients with ratios of small integers, we have,
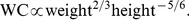
(2)


We defined A Body Shape Index (ABSI) to be proportional to the ratio of actual WC to the WC expected from the regression allometry:
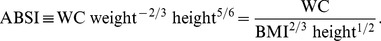
(3)


The sample mean and standard deviation of ABSI thus defined is 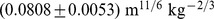.

Correlation coefficients of ABSI with height, weight, BMI and WC in the NHANES sample are shown in Table 1. It can be seen that most variability in WC reflects variability in BMI (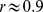) and that unlike BMI, WC also has some correlation with height (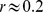), consistent with earlier findings [24]–[26]. On the other hand, ABSI shows little correlation with height, weight, or BMI (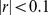). Its correlation with WC is modest (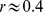), since most variability in WC is correlated with BMI and therefore excluded from ABSI.

**Table 1:** Correlations between body size and shape.

	Height	Weight	BMI	WC	ABSI
Height	1	0.452	−0.040	0.174	0.040
Weight	0.380	1	0.867	0.874	0.049
BMI	0.007	0.922	1	0.881	0.019
WC	0.163	0.908	0.918	1	0.439
ABSI	0.041	0.020	0.007	0.361	1

Correlation coefficients between height, weight, BMI, WC, and ABSI among NHANES nonpregnant adults (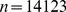). Right side (above diagonal) shows correlations of the raw values; left side (below diagonal) shows correlations of the z scores relative to age- and sex-specific means.

### Conversion to z Scores

**Figure 1:**
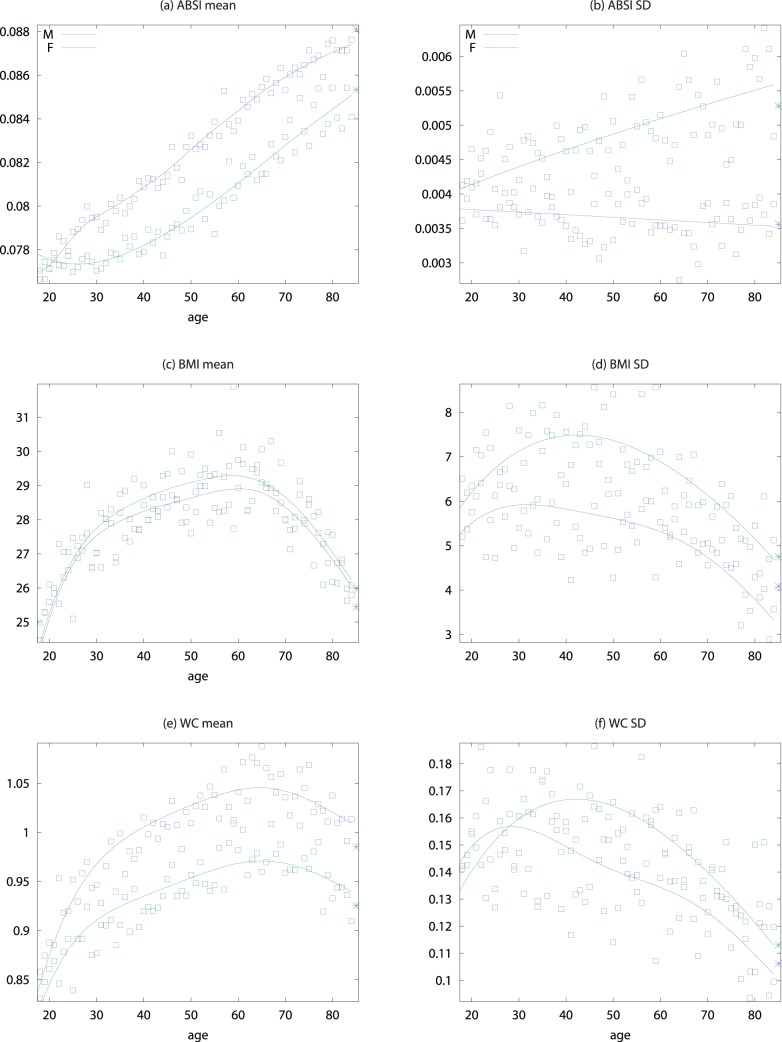
Mean and standard deviation of ABSI, BMI, and WC by age and sex. Markers show the sample quantities for each age; the smooth curves shown were used to convert values to z scores. Units are 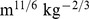 for ABSI, 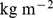 for BMI, and 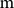 for WC.

To control for age and sex differences in mean ABSI, we entered it into proportional hazards regression for mortality as a z score:
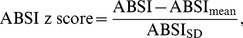
(4)where the population ABSI mean and standard deviation depend on age and sex. To estimate 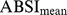 and 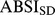, we first computed the sample mean and standard deviation for each age, separately for males and females and using the NHANES sample weights (markers in Figure 1a-b). Then we smoothed the 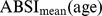 and 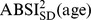 curves for each sex using Tikhonov regularization with a regularization matrix that approximates a second derivative operator and a regularization parameter chosen so that the mean square residual between the curve and the sample values, scaled by the estimated standard error of the sample values, is equal to 1 [27]. These smoothed values (curves in Figure 1) were used for converting ABSI to z scores following Eq. 4. Individuals age 85 and over (for whom the exact age was not available) were not included in the smoothing, and their ABSI values were converted to z scores using the sample mean and standard deviation (asterisks near right edges of panels in Figure 1). The age and sex specific 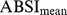 and 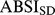 used for computing ABSI z scores are tabulated as (Table S1).

Mean ABSI increased steadily from midlife into old age (Figure 1a). Mean ABSI was consistently higher in males than females after young adulthood (Figure 1a), while the scatter in ABSI at a given age was greater in females than in males (Figure 1b). The age- and sex-specific BMI and WC means (Figure 1c,e), calculated using the same approach, showed different behavior than ABSI, falling after about age 60. Mean WC was higher in males while mean BMI was higher in females, consistent with the higher mean ABSI in males compared to females. As with ABSI, variability in BMI and WC was higher in females than in males (Figure 1d,f).

### Mortality Hazard Modeling

To quantify the association of baseline ABSI with death rate, we employed Cox proportional hazard modeling for mortality with age as the time scale [28]. In this approach, log death rate is modeled as a nonparametric function of age plus fitted coefficients that multiply the values of predictors, such as baseline ABSI. Predictors may be entered as continuous variables or discretized into two or more categories (such as quantiles of ABSI), depending on their nature and the desired model. ABSI and the other anthropometric variables (BMI, WC) were entered as z scores relative to age- and sex-specific normals, obtained as described above, to avoid confounding by age and sex differences in body size and shape.

Two types of models were employed. In one (‘unadjusted’), the anthropometric variable of interest (ABSI, BMI, or WC) was the only predictor entered. In the second (‘adjusted’), additional predictors corresponding to other known major risk factors were entered. Comparing the unadjusted and adjusted model coefficients showed to what extent the mortality risk associated with higher ABSI, BMI, or WC changed when these other risk factors are controlled for. The additional factors considered were sex, ethnicity, smoking status, presence of diabetes, blood pressure, and serum cholesterol. Sex, ethnicity, smoking status, and presence of diabetes were all entered as binary variables. Ethnicity was entered as 1 for blacks and 0 for all others, since we found that blacks had significantly elevated death rates compared to the other four ethnicities, whose death rates were not significantly different from each other. Systolic and diastolic blood pressure and total and HDL cholesterol levels were each entered as z scores relative to age- and sex-specific normals, obtained as described for ABSI. Because not all individuals with anthropometric measurements also had the other data needed for the adjusted analysis, the unadjusted analyses were run twice – once for the full sample with available anthropometry (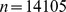) and once restricted to the sample used for the adjusted analysis (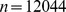).

We also determined the mortality risk associated with ABSI, BMI, and WC for subgroups of the NHANES sample, in order to test the robustness and range of applicability of coefficients determined for the entire sample. Subgroups included males and females; people younger and older than 65 yr at baseline; the three largest ethnic groupings (whites, blacks, and Mexicans); and people with BMI above and below the age- and sex-specific mean. As another check of whether these attributes impact the association with mortality, we checked the significance in the proportional hazard model of interaction terms of ABSI (BMI, WC) with sex, age, ethnicity variables (white, black, or Mexican), and BMI. To address the question of whether ABSI predicts medium-term as compared to short-term mortality, we conducted an additional analysis where the modeled follow-up period started 3 yr after the baseline, thus excluding from consideration all deaths within 3 yr of examination.

In Cox proportional hazard modeling, the relationship between hazard (here, death rate) and continuous variables, such as ABSI here, is most commonly estimated on the assumption that the logarithm of the hazard is a linear function of the variable; this yields a single regression coefficient that summarizes the strength of the relationship between the variable and log hazard. A recommended test of this linearity assumption is to fit an alternative model where the dependence of log hazard on the variable is described by a smoothing spline, with the degree of smoothing determined to optimize the Akaike Information Criterion [29], [30]. Linearity is rejected if the nonlinear terms in the fitted smoothing spline are different from zero with low p value. Our testing showed that the linearity assumption did not hold for ABSI, BMI or WC. In showing results from the models described above, we retained the linearity assumption for all three variables to facilitate comparing mortality hazards across populations and population subgroups. In separate analyses, we also fit smoothing splines to the association with mortality risk of ABSI, BMI, and WC in order to visualize it as accurately as possible. To quantify in a simpler form the nonlinear relationship between ABSI (BMI, WC) and log mortality, we also carried out analyses where risk was computed separately for each quintile of the ABSI (BMI, WC) z score, relative to the middle quintile.

A measure of the fraction of the total population mortality hazard predicted by high values of ABSI (BMI, WC) was calculated as.
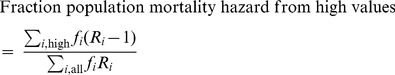
(5)where 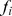 is the fraction of the population in each quantile and 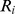 is the relative risk in each quantile. The quantiles we used for this calculation were the top 40%, middle 20%, and bottom 40%, with the top 40% regarded as the ‘high’ range included in the numerator, and risks were relative to the middle quintile. Uncertainty in this expression was approximated as being due only to uncertainty in the numerator 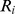.

While converting variables to z scores before entering them into a hazard regression model may be methodologically preferable given the nonlinear effects of age and sex on mean ABSI, BMI, and WC (Figure 1), we also conducted the same proportional hazard modeling using the original variables, rather than z scores, as predictors. For these analyses, sex was included as a predictor even for the unadjusted models, in order to control for the sex differences in ABSI, BMI, and WC distributions.

Proportional hazard modeling, including differential sample weighting and adjustment for the cluster survey design, was carried out using the survey package in the computer language R [31]. For all analyses, 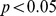 (two-tailed) was taken as the threshold for statistical significance. This corresponds to the 95% confidence interval of the exponent of a linear regression log hazard coefficient not including 1 (as would be the case under the null hypothesis that the variable has no effect on the death rate).

## Results

### Higher Mortality Hazard for Increasing ABSI

Table 2 shows the impact of ABSI z score, as a continuous variable, on death rate, along with results for BMI and WC z scores. ABSI clearly has distinct impacts on mortality compared to BMI and WC: if we model the relationship between the z scores and log mortality risk as linear, the regression coefficients imply that uniformly increasing the population ABSI by one standard deviation would result in a significant increase of 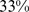 (95% confidence interval: 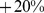-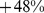), while the corresponding linear regression coefficients for BMI and WC are not significantly different from zero. Because the proportional hazard regression coefficients for ABSI, BMI, and WC showed little impact – generally shifting by less than their standard error – from either restricting the sample to those with data for the other risk factors (sex, ethnicity, smoking, diabetes, blood pressure, and serum cholesterol; middle column of Table 2) or from adjusting for the other risk factors (right column of Table 2), we carried out the analyses described below with unadjusted models.

**Table 2:** Body size and shape z scores and mortality hazard.

	Hazard ratio per SD increase		
	Unadjusted	Restricted	Adjusted
ABSI	1.33 (1.20–1.48)	1.37 (1.23–1.53)	1.30 (1.16–1.44)
BMI	0.98 (0.89–1.08)	0.98 (0.88–1.09)	0.96 (0.86–1.08)
WC	1.07 (0.98–1.16)	1.08 (0.98–1.20)	1.05 (0.94–1.17)

Results of Cox proportional hazard modeling for mortality risk with ABSI, BMI, or WC z scores taken as linear predictors. Ranges in parentheses are 95% confidence intervals. The restricted models are unadjusted but included only those people who had all the measurements required for the adjusted model. The adjusted models included as additional predictors sex, ethnicity, smoking, presence of diabetes, blood pressure, and serum cholesterol.

SD  =  standard deviation.

### Mortality Hazard from Increasing ABSI by Subgroup

Restricting our analysis to males or females within the sample does not significantly change the impact of increasing ABSI on mortality (Table 3); the change in mortality hazard per standard deviation increase in ABSI is 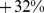 (95% confidence interval: 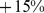
–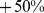) for males and 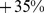 (95% confidence interval: 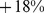
–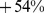) for females. Further, high ABSI predicts similar elevation of relative mortality hazard for younger (age 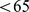 yr at baseline) and older (age 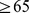 yr) individuals, with narrower confidence intervals for the older group because of their much higher absolute death rate over the follow-up period (Table 3). ABSI predicted mortality among individuals with above-mean BMI about as well as it did for individuals with below-mean BMI (Table 3). Among the three main ethnic groups in the sample, ABSI predicted mortality in both whites and blacks, while ABSI was not a significant predictor of mortality in Mexicans (Table 3).

**Table 3:** Mortality hazard by subgroup.

	Hazard ratio			
	Deaths/N	ABSI	BMI	WC
All	828/14105	1.33 (1.20–1.48)	0.98 (0.89–1.08)	1.07 (0.98–1.16)
Male	502/7133	1.32 (1.15–1.50)	0.92 (0.81–1.06)	1.00 (0.88–1.14)
Female	326/6972	1.35 (1.18–1.54)	1.04 (0.91–1.19)	1.14 (1.01–1.30)
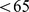 yr	213/10728	1.37 (1.12–1.69)	1.05 (0.84–1.31)	1.12 (0.91–1.39)
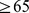 yr	615/3377	1.31 (1.18–1.45)	0.94 (0.87–1.02)	1.04 (0.96–1.12)
White	483/6709	1.43 (1.26–1.62)	1.02 (0.92–1.13)	1.12 (1.02–1.24)
Black	165/2882	1.21 (1.02–1.43)	0.68 (0.54–0.85)	0.76 (0.63–0.91)
Mexican	134/3392	1.11 (0.95–1.29)	0.78 (0.61–0.99)	0.84 (0.63–1.12)
High BMI	356/6011	1.37 (1.19–1.59)	1.20 (1.01–1.42)	1.39 (1.21–1.62)
Low BMI	472/8094	1.31 (1.12–1.51)	0.50 (0.40–0.62)	0.80 (0.64–1.00)
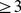 yr follow up	408/11346	1.32 (1.15–1.52)	1.01 (0.90–1.14)	1.10 (1.00–1.22)
Interaction term p-values				
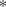 female		0.81	0.24	0.17
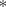 age		0.93	0.28	0.58
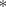 white		+, 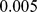	0.11	0.052
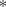 black		0.21	−, 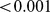	−, 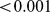
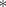 mexican		0.07	0.16	0.16
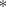 bmi		0.36	+, 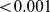	+, 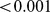

Cox proportional hazard modeling for mortality hazard ratio per unit of ABSI, BMI or WC z score (standard deviation). Ranges in parentheses are 95% confidence intervals. The sign of significant interaction terms is given. High BMI is defined as exceeding the age- and sex-specific population mean.

These conclusions from subgroup analysis were largely borne out by checking the significance of interaction terms added to the Cox proportional hazard model. ABSI
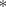
age, ABSI
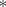
sex, and ABSI
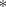
BMI interactions were not significant, confirming that high ABSI predicts mortality across these categories. By contrast, the impact of increasing BMI and WC depended strongly on BMI (Table 3), consistent with U-shaped relationships where lower weight would increase mortality at low BMI and decrease it at high BMI. Interaction with ABSI of an indicator variable for white ethnicity were significantly positive, implying that whites with high ABSI show greater relative risk elevation than other USA ethnicities.

High ABSI continued to be a significant predictor of death even when the first 3 yr of the follow-up period were excluded (Table 3), suggesting that the correlation of higher ABSI with death rate is not merely due to a propensity of acutely ill people to have high ABSI.

### Mortality Hazard by ABSI Quantile

To examine the correlation of different levels of ABSI with death rate, we stratified the population into quintiles by ABSI z score, where the middle (third) quintile included those near the population mean ABSI, and conducted proportional hazard modeling with ABSI quintiles, rather than ABSI z score, as the predictor variables, with hazard ratios expressed relative to the middle quintile. We found that people with low ABSI (first and second quintiles) had nonsignificantly decreased mortality risk relative to the middle quintile, while ABSI in the fourth and fifth quintiles was associated with progressively and significantly increased mortality risk (Table 4).

**Table 4:** Mortality by quintile.

	Hazard ratio		
Quintile	ABSI	BMI	WC
1 (lowest)	0.97 (0.69–1.37)	1.88 (1.44–2.45)	1.51 (1.12–2.03)
2	0.93 (0.64–1.35)	1.23 (0.89–1.69)	1.31 (0.92–1.87)
3 (reference)	1	1	1
4	1.46 (1.08–1.99)	1.37 (0.95–1.97)	1.30 (0.95–1.77)
5 (highest)	1.93 (1.39–2.68)	1.71 (1.22–2.39)	1.72 (1.28–2.32)

Cox proportional hazard modeling for mortality risk with ABSI, BMI or WC z score quintiles taken as the predictors. Hazard ratios are relative to the middle quintile in each case. Ranges in parentheses are 95% confidence intervals.

The between-quintile cut points are 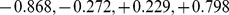 for ABSI; 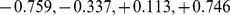 for BMI; and 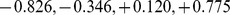 for WC.

**Figure 2:**
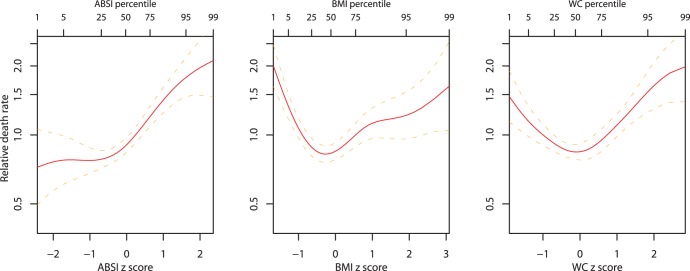
Mortality hazard by ABSI, BMI, and WC z score relative to age and sex specific normals. Estimates are from proportional hazard modeling where log mortality hazard is a smoothing-spline function in ABSI, BMI, or WC. Dashed curves show 95% confidence intervals. Corresponding population percentiles are given in the top axis; the range shown is the 1st through 99th percentiles. The vertical axis is logarithmic.

Similar analyses were conducted with BMI and WC quintiles. BMI and WC in the first quintile were both associated with significantly greater mortality hazard than for the middle quintile. Significantly increased death rate compared to the middle quintile was also seen for WC and BMI in the fifth quintile (Table 4). Comparing the excess mortality hazard from high ABSI (top two quintiles) with that posed by high BMI and WC as a fraction of the total population death rate, we found that 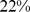 (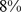
–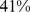) of the population mortality hazard was attributable to high ABSI, compared to 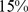 (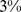
–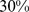) for BMI and 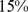 (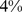
–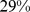) for WC. Plotting estimated mortality risk by percentile from models with ABSI, BMI, or WC as continuous predictor variables (Figure 2), with smoothing splines used to represent their nonlinear associations with mortality, gives results consistent with the quantile analyses. While risk increases progressively with increasing ABSI, BMI and WC risk is lowest near the population median and increases for both high and low values.

**Figure 3:**
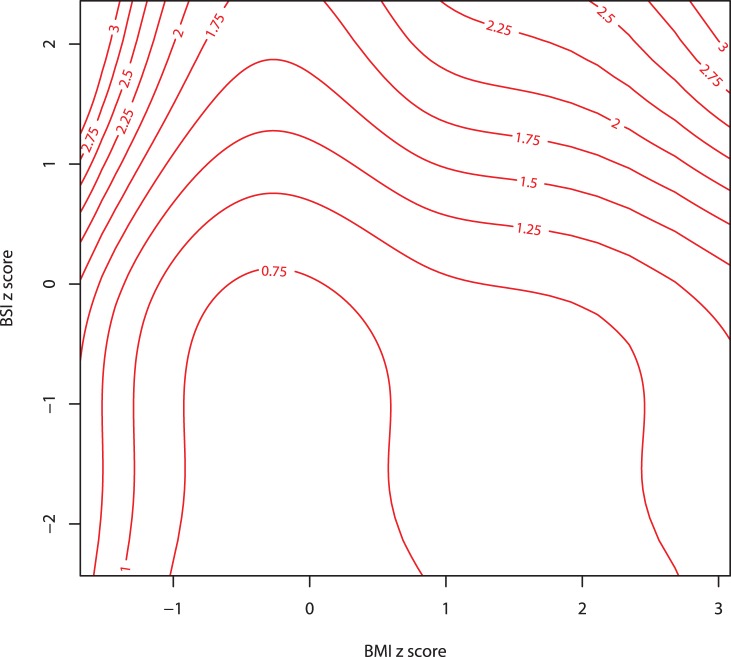
Estimated mortality hazard (relative to the population mean) by combination of BMI and ABSI z score. The ranges of BMI and ABSI shown correspond to the 1st through 99th percentiles. The contour interval is 0.25.

Figure 3 shows the estimated relative mortality risk taking both body size (BMI) and shape (ABSI) z score into account. These estimates are based on the lack of interaction we found between BMI and ABSI as predictors of mortality, so that the estimated mortality risk given both values is the product of that due to each separately (Figure 2a, b). Lowest risk is found at below-average ABSI and near average BMI (lower middle of plot), while risk elevations of above 100% are found at high ABSI and BMI either below or above average (upper left and upper right corners of plot).

**Figure 4:**
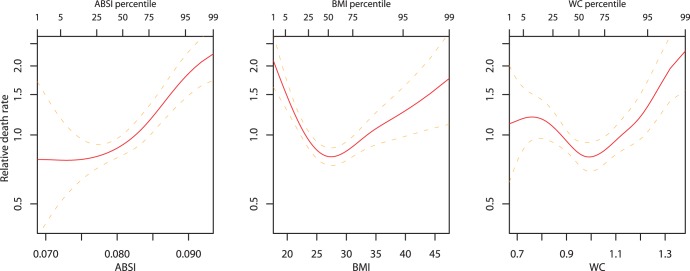
Mortality hazard by ABSI, BMI, and WC. Estimates are from proportional hazard modeling where log mortality hazard is a smoothing-spline function in ABSI, BMI, or WC. Dashed curves show 95% confidence intervals. Corresponding population percentiles are given in the top axis; the range shown is the 1st through 99th percentiles. The vertical axis is logarithmic. This is the same as Figure 2, but with ABSI, BMI, or WC, rather than their z scores, used as predictors. Units are 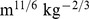 for ABSI, 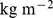 for BMI, and 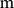 for WC.

We found that our main results continued to hold when the original variables, rather than their z scores, were used as mortality predictors. Log mortality risk increased steadily with ABSI, while decreasing for increasing BMI and WC up to values around the population median (Figure 4). The association of ABSI with mortality hazard remained after adjusting for other known risk factors (Table 5).

**Table 5:** Body size and shape and mortality hazard.

	Hazard ratio per SD increase		
	Unadjusted	Restricted	Adjusted
ABSI	1.39 (1.23–1.57)	1.44 (1.26–1.63)	1.34 (1.18–1.51)
BMI	1.00 (0.88–1.13)	0.99 (0.87–1.13)	0.97 (0.84–1.12)
WC	1.08 (0.97–1.21)	1.10 (0.97–1.25)	1.06 (0.93–1.22)

Results of Cox proportional hazard modeling for mortality risk with ABSI, BMI, or WC, as well as sex, taken as linear predictors. Ranges in parentheses are 95% confidence intervals. The restricted models are unadjusted but included only those people who had all the measurements required for the adjusted model. The adjusted models included as additional predictors ethnicity, smoking, presence of diabetes, blood pressure, and serum cholesterol.

This is the same as Table 2, but with ABSI, BMI, or WC, rather than their z scores, used as predictors.

SD  =  standard deviation. The population standard deviations used here are 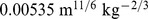 for ABSI, 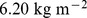 for BMI, and 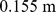 for WC.

## Discussion

The newly developed and applied ABSI is based on WC, weight and height, where high ABSI indicates that WC is higher than expected for a given height and weight and corresponds to a more central concentration of body volume. Applying ABSI along with BMI as a predictor variable separates the influence of the component of body shape measured by WC from that of body size. Our finding that higher ABSI predicts mortality hazard is thus quite analogous to the outcome of analyses which have adjusted WC for BMI without invoking ABSI. Thus, an analysis of mortality outcomes in an elderly (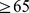 yr) USA cohort found that including both BMI and WC as continuous variables in a Cox proportional hazard model for mortality results in a direct correlation between WC and mortality and an inverse correlation between BMI and mortality [12]. In a large multination European cohort, stratifying by BMI category transformed the curve of mortality risk as a function of WC from U-shaped to more linear, similar to our curve of mortality risk as a function of ABSI quantile [14]. Our work also follows on findings that dividing WC by height increases its ability to predict cardiometabolic risk factors [32]–[35]. Some conceptual advantages of introducing ABSI are that it accounts for the sublinear increase of WC with BMI (i.e. 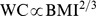) along with the nonlinear association of WC with height, and that using it instead of WC avoids inflation of regression uncertainty associated with the near collinearity of WC and BMI.

In the USA population as sampled in NHANES, ABSI predicted mortality risk across age, sex, and weight, although the ethnic difference found suggests that analysis of more cohorts is needed to delineate the limits of ABSI’s utility. A logical next step would be to investigate the association of ABSI with longer-term mortality risk, as well as its ability to predict morbidity and impaired quality of life.

What aspect of human physiology measured by ABSI accounts for its association with death rate? At a given height and weight, high ABSI may correspond to a greater fraction of visceral (abdominal) fat compared to peripheral tissue. As mentioned in the Introduction, excess visceral fat has been associated with a variety of potentially adverse metabolic changes. Equally important may be that individuals with high ABSI have a smaller fraction of mass as limb muscle; lean tissue mass and limb circumference have been shown to have strong negative correlations with mortality risk [7], [36]. Dual energy X-ray absorptimetry body composition data for most of the NHANES 1999–2004 sample is available [37], [38] and could be used to investigate ABSI’s associations with muscle, fat and bone fraction by site. For example, we found that ABSI is positively correlated to trunk fat mass as estimated from X-ray scans (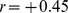 between z score of trunk fat mass adjusted for height and weight and ABSI z score) but negatively correlated with limb lean mass (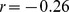), consistent with the above hypotheses; by contrast, WC has only weak associations with both trunk fat and limb lean mass after these are adjusted for height and weight (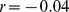 for both), suggesting that it is a less consistent indicator of changes in body shape and composition not reflected in height and weight.

We found that both low and high BMI increased the mortality hazard compared to near-median BMI (U-shaped curve for mortality hazard vs. BMI and WC in Figure 2). In the studied population, the hazard sustained by low BMI quantiles appears to be at least as great than that sustained by corresponding high BMI quantiles, consistent with the nonsignificantly negative linear regression coefficient for mortality hazard on BMI z score (Table 2). This substantial mortality hazard for low BMI held even though few (292/14105 or 2.1%) of the study population were in the WHO ‘underweight’ category of 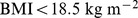. The lowest mortality hazard was for the middle quintile of both BMI and WC, although the population median was well in the WHO ‘overweight’ or ‘pre-obese’ category [39]: the 40th-60th percentile range for the sample was 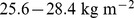, with the exact cutoffs for the middle quintile of BMI z score varying by age and sex (Figure 1c; cf. Figure 4b). Similarly, the 40th-60th percentile range of population WC was 94–101 cm for men and 88–97 cm for women, above most suggested cut-off points for higher mortality hazard [18]. These results add to many previous studies that show high population mortality hazard even in developed countries from underweight compared to overweight, particularly among the elderly and chronically ill [15], [40]–[45], supporting a rethinking of BMI-based obesity thresholds [46]. However, since high ABSI appears to identify increased mortality risk independent of BMI, it could complement either low or high BMI in risk assessment, as illustrated in Figure 3.

In addition to WC, inverse hip circumference, or waist to hip ratio, have been suggested as alternative measures of body shape that predict mortality better than BMI [18], [47]. It is theorized that gluteofemoral fat may benefit health by removing free fatty acids from the bloodstream [48]. Different studies have reached a range of conclusions about whether WC [14], [16] or waist to hip ratio [13], [17] is a better predictor of mortality; a meta-analysis of British studies found them to be equally good predictors [49]. A recent prospective analysis from Mauritius found that higher WC and lower hip circumference both correlated with greater mortality risk, while BMI did not correlate with mortality risk [50]. An analysis of an earlier NHANES cohort (NHANES III, examined 1988–1994) found that neither low nor high waist to hip ratio significantly affected mortality hazard compared to an intermediate reference level, where levels were defined by analogy with WHO obesity categories [41]. That study also found that waist to hip ratio, despite its nondimensional form, was significantly correlated to BMI (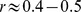), and we may hypothesize that adjusting hip circumferences or waist to hip ratios for height and weight, as done here for WC, would make them more useful as predictors of mortality hazard. The significance of hip circumference or waist to hip ratio cannot be evaluated with NHANES 1999–2004 data because hip circumference was not measured, though it is possible that adjusting WC for height and weight may indirectly provide similar information to waist to hip ratio – i.e. a wider waist for given height and weight may imply narrower hips, and vice versa. It would be of interest to compare the waist to hip ratio’s performance with that of ABSI in suitable data sets, and it may well be that ABSI can be improved as an indicator of hazardous body shape by including hip circumference in addition to height, weight and WC (which is why ABSI for now bears the indefinite article).

This prospective study does not directly address whether interventions aimed at reducing ABSI would reduce mortality risk, independent of weight change, for which large randomized controlled trials would be necessary. If ABSI does reflect malleable body shape and composition attributes, however, we may speculate that the effectiveness of weight loss interventions in improving health outcomes would be affected by how they impact WC relative to weight, since ABSI varies with the ratio 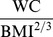. Lifestyle change that reduces ABSI, such as an exercise program that builds skeletal muscle, may yield health benefits independent of the amount of weight loss; indeed, exercise has been shown to have beneficial health impacts for obese individuals, including reductions in WC (and hence ABSI), even when weight loss does not occur [51]. Weight loss programs including either low-calorie diets or exercise can also reduce WC, along with BMI, enough to reduce ABSI [52], [53]. As other possible applications, the strong association of ABSI with mortality may be of interest to actuaries [54], and may be used as a selection criterion for enrollment in clinical trials desired to have higher power to detect mortality outcome differences with given sample size. Note that since ABSI varies over a small range (population standard deviation is of order 5%, Figure 1b), it is sensitive to the accuracy of the biometric measurements on which it is based. In particular, WC should be measured according to the NHANES protocol [22] in order to meaningfully compare ABSI with the population normals given here, even though in general the association of WC with health outcomes seems independent of the specified measurement protocol [55].

In summary, body shape, as measured by ABSI, appears to be a substantial risk factor for premature mortality in the general population derivable from basic clinical measurements. ABSI expresses the excess risk from high WC in a convenient form that is complementary to BMI and to other known risk factors.

## Supporting Information

Table S1 **This table contains the mean and standard deviation of ABSI by age and sex for NHANES 1999–2004, based on all individuals with available data (except pregnant women) and weighted to represent the larger USA population which NHANES was intended to sample.** The smoothed means and standard deviations shown were used to generate ABSI z scores. The table contains 11 space-separated columns in plain text. The layout is: Column 1: Age (85 includes those older than 85). Column 2: Number of males with available data at this age. Column 3: ABSI mean for males at the given age. Column 4: ABSI standard deviation for males at the given age. Column 5: Smoothed ABSI mean for males at the given age. Column 6: Smoothed ABSI standard deviation for males at the given age. Columns 7–11: Same as columns 2–6, but for females.
(TXT)
